# A comprehensive assessment for community-based, person-centered care for older adults

**DOI:** 10.1186/s12877-020-1502-7

**Published:** 2020-06-05

**Authors:** Eliah Aronoff-Spencer, Padideh Asgari, Tracy L. Finlayson, Joseph Gavin, Melinda Forstey, Gregory J. Norman, Ian Pierce, Carlos Ochoa, Paul Downey, Karen Becerra, Zia Agha

**Affiliations:** 1 University of California, San Diego, 9500 Gilman Drive, La Jolla, CA 92093 USA; 2 Gary and Mary West Health Institute, 10350 N. Torrey Pines Road, La Jolla, CA 92037 USA; 3 San Diego State University, 5500 Campanile Drive, San Diego, CA 92182-4162 USA; 4 Serving Seniors, 1525 Fourth Avenue, San Diego, CA 92101 USA; 5 Gary and Mary West Senior Dental Center, 1525 Fourth Avenue – second floor, San Diego, CA 92101 USA

**Keywords:** Comprehensive geriatric assessment, Oral health assessment, Person-centered care, Integrated service delivery model, Emergency department use, Hospitalization, Dental urgency

## Abstract

**Background:**

Many health and social needs can be assessed and met in community settings, where lower-cost, person-centered, preventative and proactive services predominate. This study reports on the development and implementation of a person-centered care model integrating dental, social, and health services for low-income older adults at a community dental clinic co-located within a senior wellness center.

**Methods:**

A digital comprehensive geriatric assessment (CGA) and referral system linking medical, dental, and psychosocial needs by real-time CGA-derived metrics for 996 older adults (age ≥ 60) was implemented in 2016–2018 as part of a continuous quality improvement project. This study aims to describe: 1) the development and content of a new CGA; 2) CGA implementation, workflows, triage, referrals; 3) correlations between CGA domains, and adjusted regression models, assessing associations with self-reported recent hospitalizations, emergency department (ED) visits, and clinically-assessed dental urgency.

**Results:**

The multidisciplinary team from the senior wellness and dental centers planned and implemented a CGA that included standard medical history along with validated instruments for functional status, mental health and social determinants, and added oral health. Care navigators employed the CGA with 996 older adults, and made 1139 referrals (dental = 797, care coordination = 163, social work = 90, mental health = 32). CGA dimensions correlated between oral health, medical status, depressive symptoms, isolation, and reduced quality of life (QoL). Pain, medical symptoms, isolation and depressive symptoms were associated with poorer self-reported health, while general health was most strongly correlated with lower depressive symptoms, and higher functional status and QoL. Isolation was the strongest correlate of lower QoL.

Adjusted odds ratios identified social and medical factors associated with recent hospitalization and ED visits. General and oral health were associated with dental urgency. Dental urgency was most strongly associated with general health (AOR = 1.78,95%CI [1.31, 2.43]), dental symptoms (AOR = 2.39,95%CI [1.78, 3.20]), dental pain (AOR = 2.06,95%CI [1.55–2.74]), and difficulty chewing (AOR = 2.80, 95%CI [2.09–3.76]). Dental symptoms were associated with recent ED visits (AOR = 1.61, 95%CI [1.12–2.30]) or hospitalizations (AOR = 1.47, 95%CI [1.04–2.10]).

**Conclusion:**

Community-based inter-professional care is feasible with CGAs that include medical, dental, and social factors. A person-centered care model requires coordination supported by new workflows. Real-time metrics-based triage process provided efficient means for client review and a robust process to surface needs in complex cases.

## Background

The growing population of older Americans presents a significant challenge for the United States’ (U.S.) health system. Many healthcare services remain poorly integrated and are often difficult to access, particularly for lower-income, ambulatory older adults at increased risk for a range of co-occurring medical, dental, and social problems [1–4]. Further, social services are not routinely screened or referred for in most of the healthcare system, let alone provided or integrated with the delivery of other health services. Clinical care is most costly when provided in certain medical settings, particularly on an inpatient basis in a hospital or in an emergency department (ED). In 2013, older adults over 65 were seen in EDs at rates of 12 per 100 for injury and 36 per 100 for illness reasons [5]. Many health and social needs can be assessed and met in community settings, where lower-cost, person-centered, preventative and proactive services predominate. Here we present a community-based intervention and model of person-centered care that incorporates a new digital Comprehensive Geriatric Assessment (CGA) to capture, summarize, and communicate medical, dental, functional, and social needs to triage vulnerable older adults to services within a senior wellness center.

CGAs have been used for decades to facilitate whole-person care, establish baseline awareness of patients’ needs, and determine appropriate and coordinated interventions [6]. CGAs are multidimensional and interdisciplinary in nature, and have historically encompassed a broad range of domains, including medical, psychosocial, functional, nutritional, and socioeconomic status [7]. CGAs support coordinated care and have been shown to improve outcomes in many settings by exposing critical needs for simultaneous or staged intervention [7–10]. However, historically, CGAs have concentrated primarily on medical context and long-term services and supports [7, 11], but they do not always assess social determinants, and none to our knowledge have included oral health. While other oral health assessment tools for older adults do exist (e.g., [12, 13]), many have been designed for use in long-term care residential facilities (for a review, see Chalmers and Pearson [14], and not used frequently in community-settings if they need to be administered by medical or dental providers. The new CGA tool was developed to integrate oral health with community-based medical and wellness services in a unique setting where a dental clinic was co-located with a senior wellness center and clients could be readily referred for clinical oral health services.

There are connections between oral health, chronic medical conditions and overall quality of life, morbidity and mortality have been documented, and comprehensive assessments should include oral health status and needs [15–20]. Additionally, psychosocial factors and quality of life are understood as intertwined with physical and oral health status, thus all these domains should be considered in assessments and interventions [21–25]. While a few studies have shown the benefits of an integrated dental and medical model for elder care, most of these models have been implemented in clinical venues rather than as part of person-centered care models in community settings [26–28]. The goal in this person-centered model is for the CGA to inform timely, appropriate triage and shared care planning for complex clients in a community-based setting.

Thus, the aims of the present study are threefold; first, we describe the CGA content and its development by a multidisciplinary team, for use in a community-based organization (CBO). Second, we describe the CGA implementation process, including the workflows, triage process, and referral pathways. This process used new digital technology that allowed real-time decision support and metrics-based referrals based on client responses. We show how assessment-derived metrics can help coordinate dental, social, and health services outside the healthcare system, or lead to referral for acute care when needed. Third, we examined cohort assessment data to better understand the prevalence of needs, and correlations between CGA domains. We also estimated adjusted associations between the CGA components and self-reported outcomes (recent hospitalizations and emergency department utilization), as well as clinically-assessed dental urgency.

## Methods

### Setting and participants

Serving Seniors’ Gary and Mary West Senior Wellness Center (SWC) and the Gary and Mary West Senior Dental Center (SDC) are non-profit CBOs co-located in downtown San Diego, California. Serving over 900 clients daily, the SWC provides physical activity, meals, and social, legal, care-coordination, and mental health services to low-income older adults. The SDC, launched in October 2016, is a four-chair dental clinic designed to focus on collaborative care management for underserved and vulnerable older adults, situated on the second floor of the SWC [29]. This study reports on a convenience sample of 996 new and existing SWC clients who consented for assessment and referral to services between October 1, 2016 and January 1, 2018. Age under 60 was the only exclusion criterion.

### Intervention

This Continuous Quality Improvement (CQI) project employed Community-Based Participatory Research (CBPR) [30–33] and human-centered design approaches [34, 35] to co-develop and pilot a person-centered care model linking health and wellness, dental, and social services by real-time CGA and metrics-based referral in a community-based setting. A multidisciplinary team of social workers, nurses, physicians, dentists, and care navigators met regularly over two years, with input from clients, and co-developed the CGA, with the new oral health assessment, prior to the SDC’s opening.

The clinicians and staff sought to extend, enhance and improve care quality and efficiency at the SWC. The team created and introduced a person-centered model of care to better identify and improve triage processes and care referrals. The addition of dental providers and services altered client care teams at this location and required shifting workflows and adding a pathway to dental care. This team proposed and refined workflows and informatics tools that provided preliminary metrics and rules for referral to support real-time decision-making for referrals and follow-ups. Not all clients that sought dental care were referred directly to dental services. By co-addressing all health and social needs with the CGA, the appropriate first referral could be made.

### Comprehensive geriatric assessment

The CGA captured clients’ demographics, social factors, healthcare utilization (including recent primary care visits, ED visits and hospitalizations), fall history, nutrition (hunger), health status (including medical conditions, and oral health and mental health status), medications, functional status, and quality of life (Table 1). In addition to standard medical history (conditions, medications, allergies), we included validated instruments for oral health, functional status, mental health and social determinants.

**Table 1:** Dimensions of the new Digital CGA adding oral health and social determinates to standard clinical metrics

Dimension	Number of Items	Details	References
**Demographics**	5	Age, sex, race, ethnicity, birthplace, primary language	UCLA Rand CGA*
**Social Determinates**	17	Health behaviors (smoking, drinking) education, monthly income, housing, insurance, household size, need for translation	Serving Seniors’ Universal Intake adapted from UCLA Rand CGA*
	3	Loneliness	UCLA 3-item Revised Loneliness Scale^a^*
	5	Health literacy	MICASA^b^* & Chew et al. 2008^c^*
**Nutrition**	1	Appropriate food consumption	National Health and Nutrition Examination Survey (NHANES 2012)*
	1	Food worry	The Q Database (California Department of Aging)
**Function**	13	Functional status	Vulnerable Elder’s Survey 13 (VES-13)*
	6	Fall history, hearing, vision, memory problems	Serving Seniors Universal Intake
**Quality of Life**	14	Older People’s Quality of Life-Brief	Older People Quality of Life Measure – Brief (OPQoL-B)*
**Medical**	26	Self-rated health Active Medical Problem Recent symptoms Allergies Pain	UCLA Rand CGA*
**Dental**	10	Self-rated oral health status Denture use Hygiene Behaviors Barriers to care Dental pain Perceived treatment needs	Hispanic Community Health Study/ Study of Latinos (HCHS/SOL)*
	2	Food limitation due to dental problems Saliva production	Geriatric Oral Health Assessment Index (GOHAI)*
	3	Access Barriers Utilization History Recent Symptoms	National Health and Nutrition Examination Survey (NHANES 2015)*
	1	Dental debility	Oral Health Impact Profile (OHIP)*
**Mental**	4	Mental Health History (active problems)	UCLA RAND CGA*
	9	Depression and Suicide screen	Patient Health Questionnaire (PHQ-9)*

*Validated instrument

^a^ Russell D, Peplau LA, and Cutrona CE. The revised UCLA Loneliness Scale: concurrent and discriminant validity evidence. J Pers Soc Psychol. 1980. 39 [3]: p. 472–80

^b^ Chew LD, et al. Validation of screening questions for limited health literacy in a large VA outpatient population. J Gen Intern Med, 2008. 23 [5]: p. 561–6

^c^ Available at: http://micasa.phs.ucdavis.edu/overview.html

We developed a new Oral Health Assessment (OHA) by drawing from a repository of existing oral health measures from the National Health and Nutrition Examination Survey [11, 36–38]. We queried a range of multiple dental symptoms, treatment needs, hygiene behaviors, self-reported oral health status, access barriers, and utilization patterns. There were 14 symptoms on a checklist, that ranged from mild to major concerns. These symptoms included experiencing dry mouth when eating or sleeping, issues with too much or too little saliva, bad breath or taste in mouth, sores in the mouth, bleeding gums, pain and difficulties with tasting, swallowing or chewing. Pain and functional limitations from chewing difficulty were examined separately, as they were considered more severe and urgent problems needing attention. The number of positive responses were summed, and a total score tabulated, with a possible range of 0–14. The symptom variable reflected a count of the total number of symptoms currently experienced by the client, per self-report. We explored different ways to summarize the information from the symptom checklist as there is no standard scoring, and conducted sensitivity analyses and consulted with the dental providers on the care team. Clients in this cohort scored between 0 and 9. We ultimately found that four or more reported symptoms appeared to be the most sensitive threshold, and these individuals were categorized as experiencing many dental symptoms and having greater needs.

Validated instruments for quality of life (Brief Older People’s Quality of Life Questionnaire, OPQoL-Brief) [39], depressive symptoms (Patient Health Questionnaire, PHQ-9) [40], and functional status (Vulnerable Elders Survey, VES-13) [41] served as a backbone to query psychosocial status and function. The PHQ-9 and VES-13 are widely validated measures, scored based on standard cutoff values accepted in the literature [39–42]. PHQ-9 scores greater than 5 were categorized to indicate presence of moderate to high risk for depressive symptoms (possible PHQ-9 full scale range: 0–27). VES-13 scores of 3 or higher were categorized as moderate functional limitation (possible VES-13 full scale range: 0–10). OPQOL-B did not have standard cutoff scoring, and the full scale ranges 13–65. For this study, after sensitivity analyses, we categorized the lowest quartile (those in the sample scoring 26–47) as having low QoL.

Paper versions of the assessment (*n* = 100) were first tested for time and comprehension. The finalized assessment was translated into Spanish and Mandarin using professional services. Care navigators were SWC staff that were part of the SWC care coordination team, supporting the clinicians. Care navigators conducted the CGAs with clients, reviewed the metrics, and made care referral decisions.

### Data analysis

Analyses were performed using SAS Studio 3.6 (SAS Studio Inc., Cary, North Carolina) and R Studio 3.2.5 (R Studio Inc., Boston, Massachusetts). Cohort characteristics and metrics-informed care navigators’ final referral decisions were summarized descriptively. Pearson correlations assessed associations across all metrics (*p* < .05 for two-tailed test). Odds ratios with confidence intervals were calculated for recent ED visit, hospitalization, and dental urgency models, adjusted for age, sex, race and ethnicity. Recent ED visit and recent hospitalization with an inpatient stay were each defined as within the last 6 months, and dental urgency was based on BSS assessment. Additional covariates in the models were modeled as risk factors, and included demographics (single marital status, homelessness, and living alone), medical/functional status (fair/poor self-rated general health status, falls in last year, presence of pain, medical symptoms and conditions, presence of various health conditions, hearing or memory problems, lack of primary care provider, and VES-13), dental status (more than four dental symptoms, difficulty chewing, toothache), and psychosocial factors (mental health diagnosis, major depressive disorder, suicidal ideation, feeling isolated, quality of life (QoL) based on OPQoL, depressive symptoms based on PHQ-9).

## Results

### Cohort characteristics

Cohort characteristics are shown in Table 2. Clients’ average age was 72, and 51% were men. Most clients experienced general pain (77%), 51% reported on average 2 or more medical conditions (range = 0–7), 5 prescribed medications (range = 1–20), 2 or more medical symptoms (range = 0–11) and 43% reported 4 or more dental symptoms (range = 0–9). About half (54%) were medically complex (by triage metrics) and reported more than five medications and conditions combined (including diabetes, hypertension, arthritis, and hyperlipidemia). More than two-thirds (68%) had either acute or complex dental disease, and 39% reported toothache. Half (50%) had difficulty chewing, and 14% had difficulty swallowing. Most reported good QoL (OPQoL-B = 50.2, range = 26–65), moderate functional limitation (VES-13 = 1.7, range = 0–10), and low to moderate depressive symptom scores (PHQ-9 = 3.3, range = 0–24). A significant proportion of clients were at high risk for malnutrition and food insecurity (46%). Almost half (49%) reported vision and memory problems (46%). Of those with a significant mental health diagnosis (25%), 41% reported no active treatment. About one-third (36%) reported isolation or loneliness.

**Table 2:** Cohort characteristics (*N* = 996)

Age – mean (SD)	72	(7.6)^a^
Male - n (%)	492	51
Low income 0–99% FPL	523	55
Low income 100–199% FPL	373	39
Homeless	73	8
Single/Widowed/Separated/Divorced - n (%)	775	78
Feeling isolated	78	36
Feeling left out	82	37
Have PCP - n (%)	926	93
No dental visit in last year - n (%)	520	53
Fell in the last year - n (%)	361	36
Average # of falls – mean (SD)	5.4	(2.2)^a^
Hospitalized within last 6 months - n (%)	153	16
> HS Education - n (%)	698	71
**Race**		
White/Caucasian- n (%)	578	59
Black/African American- n (%)	122	13
Asian- n (%)	191	20
Other - n (%)	59	6
**Health**		
General pain - n (%)*	767	77
Average pain score (0–10) – mean (SD)	3.8	(3.0)^a^
Toothache - n (%)	437	43
Difficulty Chewing - n (%)	486	49
Need urgent dental care - n (%)	334	33
≥ 4 Dental symptoms - n (%)	434	43
≥ 2 Medical conditions - n (%)	508	51
≥ 2 Medical symptoms - n (%)	578	58
Hypertension - n (%)	454	45
Arthritis (Rheumatoid and Osteoarthritis) - n (%)	299	30
Hyperlipidemia - n (%)	277	28
Diabetes - n (%)	205	21
CHF - n (%)	17	2
COPD - n (%)	43	4
Vision problems- n (%)	150	15
Hearing problems - n (%)	530	53
Memory problems - n (%)	400	40
≥ 1 Mental problems - n (%)	232	23
Mental diagnosis - n (%)	250	25
Mental diagnosis & in treatment - n (%)	150	15
Major depressive disorder - n (%)	128	13
Suicidal ideation - n (%)	63	6
PHQ-9 ≥ 5 depressive symptoms- n (%)	240	25
OPQoL-B, low QoL - n (%)	711	74
VES-13, low function - n (%)	374	39

a Mean (SD); *Any pain

PCP = primary care provider; HS = high school; CHF = congestive heart failure; COPD = chronic obstructive pulmonary disease; PHQ-9 = Patient Health Questionnaire; OPQoL-B = Brief Older People’s Quality of Life Questionnaire; VES-13 = Vulnerable Elders Survey

### Intake and triage

**Fig. 1:**
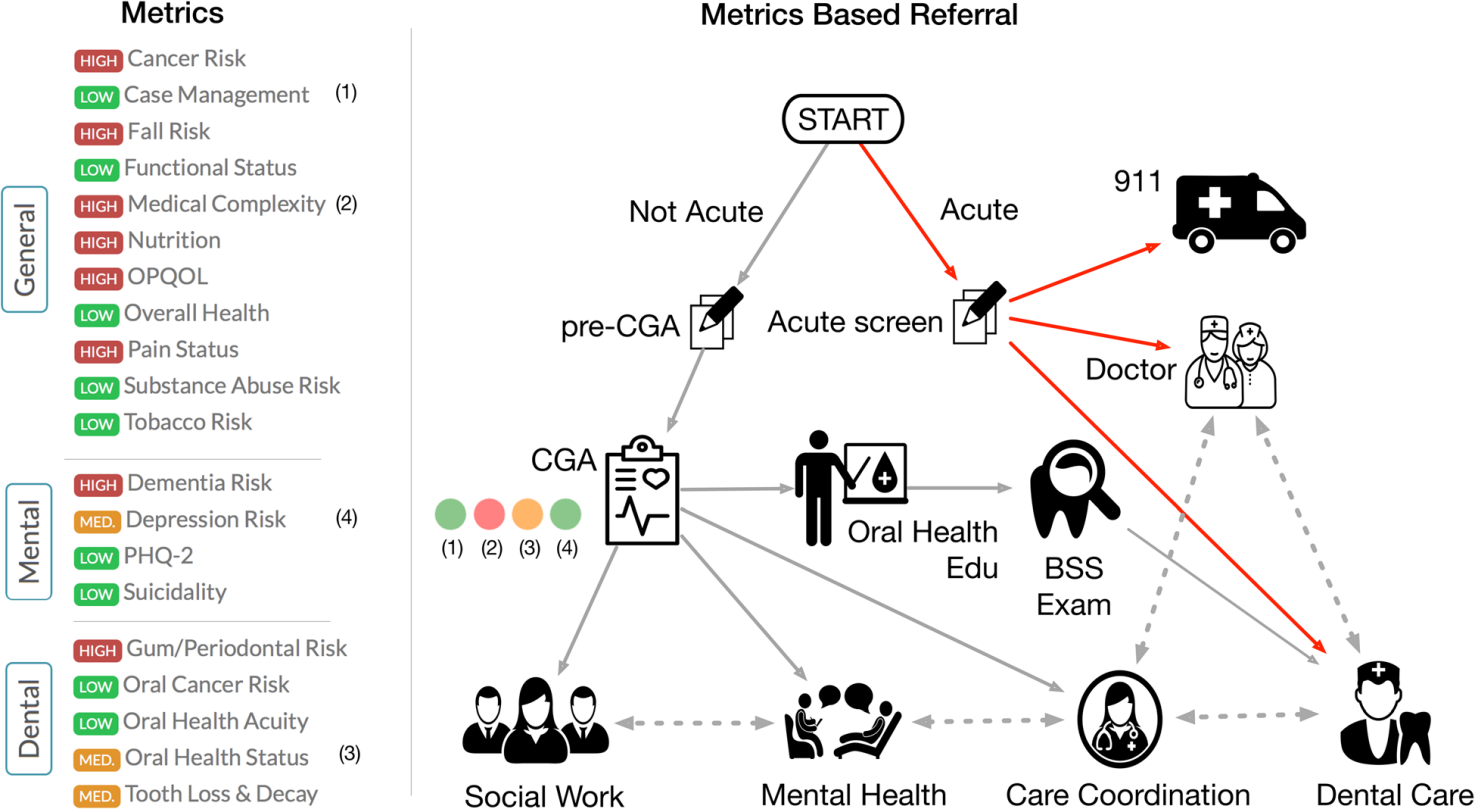
Overview of new model of person-centered care Fig. 1**a**) The digital Comprehensive Geriatric Assessment (CGA) generates biopsychosocial metrics (20 shown from the General Physical Health and Wellness, Mental Health, and Dental categories, teleoapp.com). Metrics results displayed as color-coded lights based on high (red), medium (yellow) and low (green) risk. 1**b**) The CGA facilitates appropriate referral and assignment to critical health and social services based on triage metrics (1 = Case Management need, 2 = Medical Complexity, 3 = Oral Health Status, 4 = Depression risk) and further investigation by the care navigator. Possible referral pathways are shown. BSS = Basic Screening Survey

We captured all CGA results in a prototype, shared health record which allowed data intake, metrics conversion and sharing of data with all members of the care teams. The care navigator recorded intake data and reviewed results and metrics for errors. Twenty-two metrics covering health and wellness, dental, and psychosocial needs were displayed as color-coded lights based on high (red), moderate (yellow), and low (green) risk categories (Fig. 1a). On the first pass, the care navigator could view a client list with a summary of four metrics corresponding to services referrals – medical complexity (care coordination), oral health status (dental care), depression risk/suicidality (mental health), and case management (social work) – to quickly locate key patient needs and match with available care (Fig. 1b). The care navigator then made a second pass to consider other pertinent data and metrics in the record and synthesize these for referral decisions.

Four primary referral metrics were calculated based on composite scores of multiple measures: (a) case management included “homelessness,” “lack of insurance,” “lack of Primary Care Provider (PCP),” and being “low-income” (which was defined as earning ≤199% of the U.S. federal poverty level [FPL]); (b) medical complexity included “number of medical conditions” and “number of medications,” with both defined continuously as counts, with medical conditions categorized as two or more, which was indicative of being a higher risk client with care coordination needs; (c) oral health status was comprised of: “self-rated oral health status,” “missing teeth”, experiencing more than four current dental symptoms (from a list of 14 queried), and functional impairment, including difficulty chewing and food limitation; and (d) depressive symptomology was determined by PHQ-9, with additional weight placed on suicidality (Item 9) for triage purposes.

Clients were referred to services based on the care navigator’s review of extracted metrics and key data such as symptoms, medications, and medical conditions. We designed the metrics to accelerate data review only, and during this pilot phase, referral was ultimately based on the care navigator’s judgement and client’s preference.

Staff used an Acute Clinical Screen (ACS) to assess clients with flagged medical and dental symptoms (e.g., chest pain, jaw pain, shortness of breath, bleeding, fever), and triaged them to 911, urgent medical care, or immediate dental services. Care navigators gave non-acute/well patients a brief take-home questionnaire (Pre-CGA) that included contact and basic medical information (e.g., allergies, medications, problem list, insurance, and medical contacts). Patients completed the Pre-CGA paper forms prior to the first appointment. The care navigator collected and entered clients’ Pre-CGA information, completed the assessment, and referred them to services in the same appointment. Clients with one yellow or red light were typically referred only to that service unless the client requested other services. Clients with 2 or more red lights were referred for simultaneous care, based on their most urgent need. Clients with 1 or more yellow lights were referred based on urgency and navigator’s judgement, and clients with 4 green lights seeking dental care were assigned to dental “usual-care” (described in the next section).

### Integrated dental and health workflow

Clients referred to care coordination were assessed by a registered nurse for active medical symptoms, conditions, and establishment of an appropriate medical home. The nurse also provided clients with health education, nursing care, and helped them create personal health action-plans. For clients referred to case management, social workers provided assistance with housing, legal matters, insurance eligibility, and individual and group counseling. Clients referred to mental health received acute assessments (e.g., for suicidality), acute therapy, and medication reconciliation from either the center’s psychiatric nurse or an outside provider.

Non-acute clients referred to dental care participated in a mandatory hour-long oral health education (OHE) course, (developed by Oral Health America; which has now ceased operations, but oral health resources for older adults are still available at https://www.toothwisdom.org/) followed by a Basic Screening Survey (BSS) both administered by a registered dental hygienist (RDH) prior to the client’s first dental appointment. The OHE covered topics on older adults’ oral health and common chronic conditions and provided hygiene demonstrations. We used the BSS, a brief visual exam conducted by RDH, to determine dental urgency and needs as “ground-truth,” compared to self-reported data, and to prioritize scheduling clients for dental care. We followed the standard BSS procedures for screening older adults, using the standard American Association of State and Territorial Dental Directors (ASTDD) Toolkit for Older Adults [43]. Based on the BSS, the RDH determines if a client should be seen urgently (immediately/as soon as possible, or within the next 2 weeks), soon (within weeks), or no obvious problem or dental need, and the client can continue their regular care pattern for their next check-up.

### Referral outcomes

Of 1335 clients engaged, 1012 completed a pre-CGA and 996 completed a CGA without missing responses. Of 1335 clients, 22 reported acute conditions (e.g. active chest pain, fever, severe jaw pain) and completed the ACS. Of these, 9 were referred to PCP/911 and 13 directly to urgent dental care (Table 3). CGA average time to completion was 52 min (range = 34–68). There were no significant differences in response or completion time between English, Spanish, and Mandarin speakers or written versus digital format. Of the clients completing the CGA, 158 declined services or were lost to follow-up and 52 new clients required only nutrition services.

**Table 3:** Referrals and Services for *n* = 996 completed CGAs. * N listed for those who completed services

Referrals	Identified with Need	Referred	Received (completed) Services	Referral Outcomes	N*
**Dental**	949 (95%)	901	700 (401)	Preventative Periodontics Other Restorative Prosthetics/Dentures Oral Surgery Adjunctive Endodontics	598 440 439 249 217 202 201 49
**Care Coordination**	690 (69%)	172	116 (116)	High Medical complexity/Active symptoms Connection to PCP/Medical Home Enrolled in Dental Lifeline Network program Assistance with eye exam/eyeglasses Medication reconciliation Medical equipment Assistance with hearing aids Smoking cessation	59 49 41 12 6 5 4 3
**Case Management Referrals**	230 (23%)	100	68 (68)	Assistance with health insurance Housing Income assistance Assistance with caregiving Transportation Assistance with utilities Food stamps Employment	32 30 9 5 5 3 3 2
**Mental Health Referrals**	102 (10%)	33	30 (29)	Linkage to mental health services in community Serving Seniors support services (counseling, support group) Behavioral issues/non-compliance	11 9 8

Please see Supplemental Figure 1 for a diagram of these referral pathways and outcomes

Care navigators made 1210 referrals for 996 clients (dental = 905, care coordination =172, social work = 100, mental health = 33). Of those, 701 needed only a single referral, and 225 received multiple referrals (maximum 4 referrals). The majority of single referrals (683; 98%) were for dental services. Nine clients were referred only to care coordination, 6 to case management and 2 for mental health services. Two hundred twenty clients were referred to dental and at least one more service.

There were no instances of acute medical or psychiatric issues during dental care. Almost all clients referred to dental services completed the OHE and BSS segments, with no-show rates of < 1%. Total no-show rate for dental care was 5.7%. Table 3 summarizes the distribution of services received for each referral (and see Supplemental Figure 1 for a diagram of these referral pathways and outcomes). For dental care, the most common services were preventative (598), periodontics (440) and prosthetics/dentures (217). The most common care coordination services were for medical complexity or management of specific symptoms (59), connection to medical home (46) and enrollment in external services such as specialty dental or medical care (41). Case management services were primarily for health insurance enrollment (32), housing (30) and income assistance (9). Mental health services included linkage to care (11) or counseling or support at Serving Seniors (9). Eight clients were referred to mental health for behavioral or non-compliance issues with the dental center.

### Correlations

**Fig. 2:**
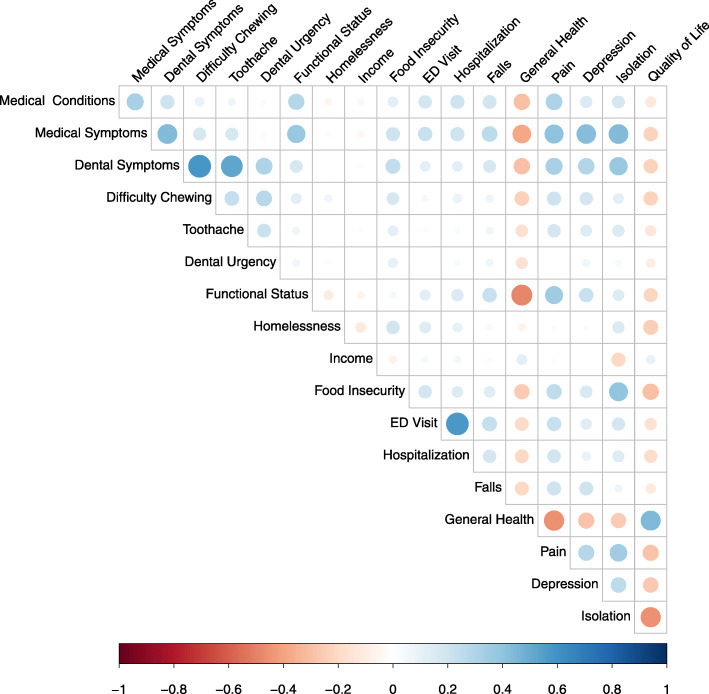
Correlation matrix of CGA key metrics. All *p* values < 0.05; Blue indicates positive and red negative correlations

Correlation analysis of CGA data (Fig. 2) showed significant relationships between the CGA dimensions (all *p*-values < 0.05). Pain was strongly negatively correlated with general health (− 0.49) and QoL (− 0.32), and positively correlated with depression risk (0.41) and lower functional status (0.43). Self-reported general health was the strongest positive correlate of QoL (0.47) and lower general health was strongly correlated with lower functional status (.53). Number of medical conditions was significantly associated with lower self-reported health (− 0.34) and QoL (− 0.18), correlated with depression (0.26), and strongly correlated with functional limitation (0.35). Medical symptoms were negatively correlated with general heath (− 0.41) and QoL (− 0.26) and associated with depressive symptoms (0.54), low functional status (0.43), and fall risk (0.30). Dental symptoms were negatively correlated with self-rated health (− 0.30) and QoL (− 0.24) and strongly correlated with depressive symptoms (0.43). Dental symptoms were significantly correlated with dental pain (0.54), food limitation (0.21), and difficulty chewing (0.59). Finally, Isolation was associated with medical (0.32) and dental (0.23) symptoms and functional limitation (0.19), strongly correlated with poor general health (− 0.29), pain (0.35) and depressive symptoms (0.43), and was the strongest correlate of low QoL (− 0.50).

### Odds ratios

Table 4 summarizes the adjusted odds ratios (AOR) and 95% confidence intervals (CI) for recent ED visits, hospitalizations, and dental urgency. ED visits were most strongly associated with homelessness (AOR = 3.18, 95% CI [1.59, 6.39]), lack of PCP (AOR = 2.43, 95% CI [1.00, 5.90]), recent falls (AOR = 2.88, 95% CI [2.01, 4.15]) and key specific medical conditions such as chronic obstructive pulmonary disease (COPD) (AOR = 3.05, 95% CI [1.45, 6.44]) and congestive heart failure (CHF) (AOR = 8.86, 95% CI [2.39, 32.80]). Those with moderate to severe depressive symptoms (AOR = 2.36, 95% CI [1.59, 3.50]) or lower QoL (AOR = 1.96, 95% CI [1.32, 2.88]) were approximately 2 times more likely to have had an ED visit within 6 months.

**Table 4:** Adjusted Odds Ratios

Predictors	Recent ED Visit			Recent Hospitalization			Dental Urgency		
	AOR^**a**^	(95% CI)		AOR ^a^	(95% CI)		AOR ^a^	(95% CI)	
Marital Status (single)	2.27	(1.36	3.79)	1.76	(1.07	2.89)	1.48	(1.02	2.15)
Homelessness	3.18	(1.59	6.39)	2.61	(1.48	4.62)	–	–	–
Live alone	1.80	(1.21	2.67)	–	–	–	–	–	–
**Medical/Function**									
Poor self-rated general health	2.04	(1.40	2.96)	2.52	(1.76	3.59)	1.78	(1.31	2.43)
Falls	2.88	(2.01	4.15)	2.21	(1.55	3.15)	–	–	–
Pain (≥1)	1.58	(1.00	2.51)	1.92	(1.18	3.11)	–	–	–
Pain (≥5)	1.40	(0.98	2.00)	1.69	(1.18	2.41)	–	–	–
Medical Symptoms (≥2)	2.03	(1.40	2.95)	1.69	(1.16	2.44)	–	–	–
Medical Conditions (≥2)	1.85	(1.29	2.65)	2.14	(1.49	3.09)	–	–	–
Arthritis	1.81	(1.26	2.59)	1.91	(1.33	2.74)	–	–	–
Hypertension	1.54	(1.09	2.19)	2.04	(1.43	2.90)	–	–	–
Hyperlipidemia	1.47	(1.01	2.13)	–	–	–	–	–	–
COPD	3.05	(1.45	6.44)	5.05	(2.70	9.42)	–	–	–
CHF	8.86	(2.39	32.80)	6.94	(2.61	18.46)	–	–	–
Hearing Problems	1.84	(1.28	2.63)	1.80	(1.26	2.56)	–	–	–
Memory Problems	–	–	–	1.84	(1.29	2.62)	–	–	–
Lack of PCP	2.43	(1.00	5.89)	3.04	(1.09	8.51)	–	–	–
VES-13	1.67	(1.15	2.42)	2.21	(1.53	3.21)	1.40	(1.03	1.91)
Dental Symptoms (≥4)	1.61	(1.12	2.30)	1.47	(1.04	2.10)	2.39	(1.78	3.20)
Difficulty Chewing	–	–	–	1.46	(1.03	2.08)	2.80	(2.09	3.76)
Toothache	–	–	–	–	–	–	2.06	(1.55	2.74)
Mental Health Diagnosis	1.88	(1.26	2.80)	1.59	(1.07	2.36)		–	–
Major Depressive Disorder	1.76	(1.08	2.87)	–	–	–	–	–	–
Suicidal Ideation	2.04	(1.01	4.10)	2.03	(1.11	3.72)		–	–
Feeling Isolated	–	–	–	–	–	–	–	–	–
QoL	1.95	(1.32	2.88)	2.42	(1.67	3.51)	–	–	–
PHQ-9	2.36	(1.59	3.50)	1.97	(1.34	2.89)	–	–	–

^a^ Adjusted for age, sex, race and ethnicity

-- *p* value > 0.05

*CHF* congestive heart failure, COPD chronic obstructive pulmonary disease, *PCP* primary care provider; *VES-13* Vulnerable Elders Survey, *QoL* quality of life, based on Brief Older People’s Quality of Life, *PHQ-9* Patient Health Questionnaire

For recent hospitalizations, the most strongly associated with homelessness (AOR = 2.61, 95% CI [1.48, 4.62]), lack of PCP (AOR = 3.04, 95% CI [1.09, 8.51]), poor self-rated general health (AOR = 2.52, 95% CI [1.76, 3.59]), hypertension (AOR = 2.04, 95% CI [1.43, 2.90]), COPD (AOR = 5.05, 95% CI [2.70, 9.42]), CHF (AOR = 6.94, 95% CI [2.61, 18.46]), low functional status (AOR = 2.21, 95% CI [1.53, 3.21]), active depression (AOR = 1.97, 95% CI [1.34, 2.89]), and low QoL (AOR = 2.42, 95% CI [1.67, 3.51]).

Dental urgency was most strongly associated with self-rated general health (AOR = 1.78, 95% CI [1.31, 2.43]), the presence of 4 or more dental symptoms (AOR = 2.39, 95% CI [1.78, 3.20]), the presence of dental pain (AOR = 2.06, 95% CI [1.55–2.74]), and difficulty chewing (AOR = 2.80, 95% CI [2.09–3.76]). Those with more than 4 dental symptoms were also more likely to have had recent ED visits (AOR 1.61, 95% CI [1.12–2.30]) or hospitalizations (AOR 1.47, 95% CI [1.04–2.1]).

## Discussion

A broader CGA that includes oral health provides a more complete assessment of health for older adults. This community-based model of person-centered care demonstrated the feasibility of bridging dental, health and social services for older adults through real-time comprehensive assessment and referral. Although the simple triage metrics used in this study could speed data review for those with multiple urgent needs or a combination of moderate and high-risk on certain metrics, further investigation by the care navigator was critical to determine the true urgency and appropriateness of referral. This supports the combination of digital assistance and human assessment, sometimes referred to as “human-technology teamwork” [44] and showcases the need to create multi-disciplinary care teams. The ongoing accrual of referral data provides the opportunity to refine these metrics in future studies.

The prevalence and tight coupling of the CGA dimensions points to the complexity of health among ambulatory older adults. Analysis of the metrics derived from the CGA paints a holistic picture of the cohort and confirmed the significant medical and dental disease burdens and barriers to care faced by clients. This illustrates not just the need for more dental service provision to this population, but the opportunity to integrate with other health and wellness providers (e.g. dentists and social workers). To this point, there have been numerous recent calls for increased integration of services and for comprehensive care to include oral health [45] and to develop team-based approaches. A recent literature review projected a primary care workforce shortage in primary care in community-based settings that is prepared to care for older adults, and that new models that can support collaboration across all caregivers on care teams are needed [46]. Dentists are frequently omitted from these types of care teams and lack sufficient training in geriatrics [47], but there is recognition among dental providers that training support is needed, so that they can care for older adult patients that may transition from being independent to becoming more dependent [48].

This cohort represents an ambulatory population, and a significant number were medically complex and economically vulnerable. Though almost all reported active care from a PCP, more than half had not seen a dentist in the past year, likely due to current differences in health and dental coverage within Medicare and Medicaid, and consistent with the historic U.S. medical-dental divide in health service delivery [49]. Though common social risk factors underlie both medical and dental problems, these dimensions are infrequently assessed together to provide complete information about client needs, and this study demonstrated the value of a CGA that assessed these dimensions together. The current general dental provider workforce lacks geriatric training and are ill-prepared to handle older adults with complex healthcare needs. While there are suggested models for dentists being trained today to include geriatric patient needs [50], there is still a current access problem for community-dwelling, lower-income older adult patients that extends beyond financial barriers. These non-financial barriers to providing person-centered care for older adults remain pervasive throughout the healthcare system, with all types of providers. Challenges also remain with integrating health and social services.

Dental services were a newly offered service at the time of this study and were desired by many clients. Co-locating free and low-cost dental services in a wellness center also eliminated some traditional dental care access barriers, contributing to the very low no-show rate (< 6%) which is far lower than typical for any dental practice [51]. We recognize that this study occurred as part of quality improvement in a unique setting and may not be easily replicable. However, there are still transferrable lessons learned here for building a system that bridges the medical, dental, and social divides. The team-based approach described here grew from collaboration of multi-disciplinary stakeholders identifying the components needed in the CGA and worked together on implementing the workflows. This approach enabled the comprehensive assessment and triage processes to be deployed.

Medical-dental collaborations that include dental providers on primary care teams to enhance access to preventive oral health care have had variable impact [52]. It is challenging to form effective, highly functional multi-disciplinary teams [53], but this mode of person-centered, interprofessional team-based collaborative care is the model of the future. Co-location alone is not sufficient without shared tools that operate in real-time and commitment to integrated workflows to support real collaboration across providers on the team. This approach also positions the team to focus on prevention efforts and wellness, especially after immediate needs are addressed. There is guidance available for creating patient-centered medical homes and caring for medically compromised older adults [54]. However, there are fewer examples and less guidance available for person-centered models of care in community settings.

Our results also add to the limited but growing literature that emphasizes the importance of addressing the oral health status of seniors as part of overall health and well-being. Poor oral health has been associated with functional status limitations among older adults in other studies [55]. Similarly, we found correlations among the CGA health metrics which highlighted key factors associated with QoL, depression, and functional status. Of these, pain, medical symptoms, isolation and depression appeared to be the strongest predictors of poorer self-reported health, while general health was most strongly correlated with lower depression, higher functional status and QoL. Isolation was the strongest correlate of lower QoL. Further, adjusted odds ratios for CGA metrics were strongly associated with key clinical outcomes ranging from emergency department visits and recent hospitalizations to fall risk and dental urgency. Our findings show the potential of CGAs to predict needs, urgency and outcomes in this population, with implications for targeted preventive and more cost-effective efforts.

## Conclusions

Community-based inter-professional care is feasible, though it requires collaborative planning and new workflows to implement in-depth client evaluation and metrics-based referrals. A real-time metrics-based triage process that included medical, dental, and social factors in this new CGA provided both an efficient means for client review and a robust process to surface needs in complex cases.

## Supplementary information

**Additional file 1: Supplemental Figure 1.** Referral pathways and outcomes. For 1335 clients screened, 1012 completed a pre-CGA and 1000 completed a CGA. For these clients, 901 dental referrals were made for services including restorative, periodontics, dentures, oral surgery and endodontics. One hundred seventy-two referrals were made for medical care-coordination, 90 for case management/social work and 32 for mental health.

## Data Availability

The datasets generated and/or analyzed in this study are not publicly available as the datasets contain confidential patient data. The data that support the findings of this study are available from West Health Institute, but restrictions apply to the availability of these data, which were used under a data sharing agreement for the current study, and so are not publicly available.

## Abbreviations

ACS: Acute clinical screen
AOR: Adjusted odds ratio
ASTDD: American association of state and territorial dental directors
BSS: Basic screening survey
CBO: Community based organization
CBPR: Community-based participatory research
CGA: Comprehensive geriatric assessment
CHF: Congestive heart failure
CI: Confidence interval
COPD: Chronic obstructive pulmonary disease
CQI: Continuous quality improvement
ED: Emergency department
FPL: Federal poverty level
OHA: Oral health assessment
OHE: Oral health education
OPQoL-Brief: Brief older people’s quality of life questionnaire
OR: Odds ratio
PCP: Primary care provider
PHQ-9: Patient health questionnaire
QoL: Quality of life
RDH: Registered dental hygienist
SDC: Gary and Mary west senior dental center
SWC: Serving seniors’ Gary and Mary west senior wellness center
U.S.: United States
VES-13: Vulnerable elders survey
