# Supplementary material for: A comprehensive assessment for community-based, person-centered care for older adults
Source: BMC Geriatr. 2020 Jun 5;20:193. doi: 10.1186/s12877-020-1502-7 (PMC7275322; doi:10.1186/s12877-020-1502-7)
Supplement: Supplementary file 1 — Additional file 1: Supplemental Figure 1. Referral pathways and outcomes. For 1335 clients screened, 1012 completed a pre-CGA and 1000 completed a CGA. For these clients, 901 dental referrals were made for services including restorative, periodontics, dentures, oral surgery and endodontics. One hundred seventy-two referrals were made for medical care-coordination, 90 for case management/social work and 32 for mental health. [file 12877_2020_1502_MOESM1_ESM.docx]

**
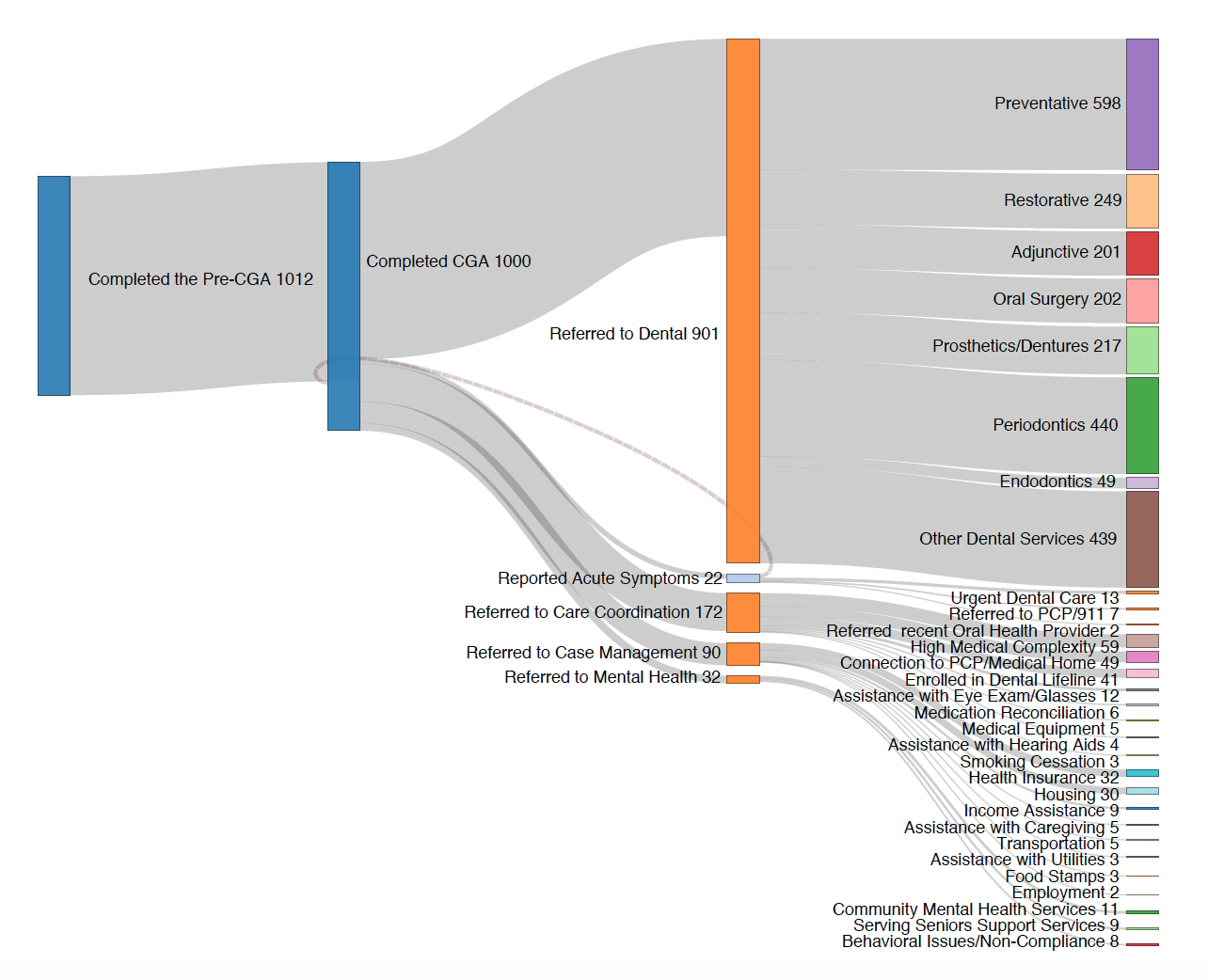
**

**Supplemental Figure 1. Referral pathways and outcomes.**

For 1335 clients screened, 1012 completed a pre-CGA and 1000 completed a CGA. For these clients, 901 dental referrals were made for services including restorative, periodontics, dentures, oral surgery and endodontics. 172 referrals were made for medical care-coordination, 90 for case management/social work and 32 for mental health.
